# Early Differentiation of Prostatic Intraepithelial Neoplasia *via* Multiparametric MRI Combined with PSA-derived Parameters

**DOI:** 10.2174/0115734056451678260513153242

**Published:** 2026-05-24

**Authors:** Guannan Zou, Jingyan Liu, Huanhuan Zhong, Heng Zhang, Yuhui Zhu, Nanai Xie, Minghua Wang, Xingzhi Li, Wanling Ma

**Affiliations:** 1 Department of Radiology, The Second Affiliated Hospital, School of Medicine, The Chinese University of Hong Kong, Shenzhen & Longgang District People’s Hospital of Shenzhen, Shenzhen,China; 2 Department of Emergency, The Second Affiliated Hospital, School of Medicine, The Chinese University of Hong Kong, Shenzhen & Longgang District People’s Hospital of Shenzhen, Shenzhen,China; 3 Department of Pathology, The Second Affiliated Hospital, School of Medicine, The Chinese University of Hong Kong, Shenzhen & Longgang District People’s Hospital of Shenzhen, Shenzhen,China; 4 Department of Urology, The Second Affiliated Hospital, School of Medicine, The Chinese University of Hong Kong, Shenzhen & Longgang District People’s Hospital of Shenzhen, Shenzhen,China

**Keywords:** Multiparametric MRI, Apparent diffusion coefficients, Prostatic intraepithelial neoplasia, Benign prostatic hyperplasia, Prostate-specific antigen, mpMRI

## Abstract

**Background:**

Prostatic intraepithelial neoplasia (PIN) is a precancerous lesion. Early differentiation of PIN is very important for the prevention of PIN occurrence and malignization.

**Objectives:**

To differentiate prostatic intraepithelial neoplasia (PIN) *via* multiparametric MRI (mpMRI) combined with prostate-specific antigen (PSA)-derived parameters.

**Materials and Methods:**

From March 2022 to June 2024, 47 males, including 39 with benign prostatic hyperplasia (BPH) and 8 with PIN, were enrolled. All patients underwent prostatic mpMRI. The apparent diffusion coefficients (ADC), *K*^trans^, *k*_ep_, *v*_e_, initial area under curve (iAUC), and PI-RADS score were derived from mpMRI. The parameters between PIN and BPH were compared *via* Student's *t* test or Mann-Whitney *U* test. Receiver operating characteristic (ROC) curves were used to evaluate the diagnostic efficacy.

**Results:**

The ADC value of PIN was significantly greater than that of BPH (0.912 *vs.* 0.806 × 10^-3^ mm^2^/s, *P*=0.035). The tPSA, fPSA, and PSA density (PSAD) of PIN were significantly lower than those of BPH (6.715 *vs.* 11.640 ng/mL, *P*=0.008; 1.427 *vs.* 2.109 ng/mL, *P*=0.042; 0.095 *vs.* 0.158 ng/mL^2^, *P*=0.010; respectively). The AUC of tPSA was slightly greater than those of PSAD, ADC, and fPSA (0.801 > 0.792 > 0.724 > 0.716) in differentiating PIN from BPH. When the tPSA was ≤ 8.47 ng/mL, the highest sensitivity (87.5%) and negative predictive value (96.8%) were observed in differentiating PIN from BPH. When the ADC and PSA-derived parameters were combined, the highest specificity (94.9%) was acquired in differentiating PIN from BPH. ADC (OR=0.120, *P* = 0.043) and PSAD (OR=0.056, *P* = 0.005) were independent risk predictors for differentiating PIN from BPH.

**Discussion:**

ADC derived from mpMRI combined with PSA-derived parameters exhibit enormous potential for differentiating PIN from BPH.

**Conclusion:**

ADC and PSA-derived parameters can distinguish PIN from BPH. The ADC value combined with tPSA can achieve the highest diagnostic efficiency in detecting PIN. The ADC may function as a potential biomarker to differentiate precancerous lesions of the prostate.

## 1 INTRODUCTION

Prostate cancer (PCa) and benign prostatic hyperplasia (BPH) are the most common urogenital diseases in elderly men. PCa is the second most common tumour and the fifth leading cause of tumour-related death in males worldwide [1]. To promote the early diagnosis and treatment of PCa, the timely detection of precancerous lesions or progression of carcinogenesis is highly important [2, 3]. Prostatic intraepi-thelial neoplasia (PIN) is currently and generally recognized as a precancerous lesion [4] that may precede clinically detected PCa by several decades [5, 6]; most patients with PIN develop malignancy within this timeframe [5]. Owing to its close association with PCa, PIN can provide early detection as well as prognostic information. PINs are classified into lower-grade PIN (LGPIN) and higher-grade PIN (HGPIN) lesions [7]. In patients with HGPIN, especially when such cases are associated with abnormal digital rectal examination (DRE) or elevated prostate-specific antigen (PSA) and/or transrectal ultrasound (TRUS), the morbidity of subsequent PCa is approximately 35-100% on repeat biopsy [8-12]. The highest correlation has been detected between HGPIN and subsequent PCa when the PSA is > 10 ng/mL [12]. However, the probability of subsequent cancer seems to be markedly different in LGPIN patients. The morbidity of subsequent PCa in LGPIN patients is zero when the PSA is < 4 ng/mL; however, this incidence is 10.7% when the PSA is located 4 to 10 ng/mL and 42.8% that is nearly as high as that in the HGPIN when the PSA is > 10 ng/mL [12]. LGPIN and HGPIN may be the chronological steps in the continuous progression of prostatic carcinogenesis [13]. Radiation therapy and androgen deprivation therapy decrease the morbidity and degree of PIN and can lead to regression of HGPIN [14, 15]. Therefore, the early detection of PIN is very important for the prevention of PIN occurrence and malignant transformation.

Serum PSA may be an important factor to consider when managing patients with PIN, especially HGPIN [9]. However, the relationship between the PSA level and PIN remains controversial. PIN alone does not seem to increase the PSA level [5]. PIN with intermediate-high PSA levels between those of BPH and PCa may be explained by concomitant hyperplasia or, most likely, by the minimal cancer present [16]. Owing to the low specificity of PSA, an evaluation of its levels may increase the risk of overdiagnosing PIN as PCa and result in an unnecessary prostate biopsy [17]. PSA derivatives, including PSA density (PASD), free PSA (fPSA), and free/total PSA (f/tPSA), can aid total PSA (tPSA) to improve the accuracy of PCa identification and avoid unnecessary biopsy [17, 18]. PASD [= tPSA/prostate volume (PV)] can increase confidence in detecting early PCa because it decreases the impact of enlarged PVs [19]. However, several authors have reported that the PIN volume is not associated with PSAD and that PSAD does not aid in strategy decisions in patients with PIN [5, 12, 16]. Because fPSA is increased in BPH patients and decreased in PCa patients, research on fPSA may be critical, especially for distinguishing between BPH patients and PCa patients [20]. The use of f/tPSA is considered valid for identifying PCa patients with PSA between 4 and 10ng/mL (the diagnostic grey zone), and the higher the f/tPSA is, the lower the PCa risk is [21].

Currently, many studies on PIN are conducted from a pathological perspective [7]. However, the imaging features of this precancerous lesion remain unclear. Recently, several studies [22-29] have evaluated the role of magnetic resonance spectroscopic imaging (MRSI), multiparametric magnetic resonance imaging (mpMRI), and magnetic resonance elastography (MRE) in the detection and characterization of PIN. mpMRI usually includes T2-weighted imaging (T_2_WI), diffusion-weighted imaging (DWI), and dynamic contrast-enhanced (DCE) MRI. Combining these sequences allows the simultaneous evaluation of different tissue properties, such as the prostatic anatomic zone, the microstructure, and the vascularization rate of prostatic neoplasms [25-29]. Thus, the present study aims to evaluate the diagnostic value of mpMRI united with PSA-derived parameters for differentiating PIN from BPH.

## 2 MATERIALS AND METHODS

### 2.1 Patient

Ethical approval of this prospective study was obtained from the Ethics Committee Board of our hospital, and informed consent was acquired from all participants. Between March 2022 and June 2024, 132 subjects with elevated PSA levels and clinically suspicious PCa were considered for inclusion in this study. Inclusion criteria include: (a) a histologically confirmed diagnosis of BPH or PIN; (b) no contraindications to gadolinium contrast agent and MRI; and (c) no treatment or surgery before MRI examination. Exclusion criteria include: (a) without pathological confirmation; (b) histologically confirmed diagnosis other than BPH or PIN; (c) loss of follow-up; and (d) with contraindications to gadolinium contrast agent and MRI. A total of 85 patients were eliminated owing to various reasons. The recruitment process was demonstrated in Fig. (**1**). Ultimately, 47 males with histopathological results on initial biopsy (age range: 37‒81 years, mean age: 66.7 ± 10.4 years) were enrolled in the present study. Among these patients, 39 were confirmed to have BPH, and 8 had PIN. All subjects in the final research cohort performed conventional prostatic MRI combined with DWI and DCE-MRI within one week before 12-core TRUS-guided biopsies.

### 2.2 MRI Examination Protocols

All subjects were examined on a 3.0 T MR scanner (MAGNETOM Prisma, Siemens Healthcare, Erlangen, Germany) with pelvic phased array coils. Routine prostatic MRI examination protocols consisted of transversal T1-weighted imaging (T_1_WI) and high-resolution T_2_WI without fat saturation in the coronal, transversal, and sagittal planes. Transversal DWI echo-planar imaging was employed with b values of 50, 500, 1000, and 1500 s/mm^2^. A precontrast variable flip angle T1-mapping sequence was performed by using 3D volume interpolated body examination (3D-VIBE) sequences with flip angles of 2°, 5°, and 10°. Then, DCE-MRI examination was performed by using fast 3D-VIBE sequences. The DCE-MRI examination time was 332 seconds, and a total of 35 phases were acquired, with 9.5 seconds for each phase. Three precontrast phases were acquired before high-pressure bolus injection, followed by the administration of 0.1mmol/kg of Gd-DTPA (Omniscan; GE Healthcare Co., Ltd., Shanghai, China) at a rate of 2.5 mL/s, followed by a 20-mL saline flush. The elaborated sequence parameters are shown in Table **1**.

According to the Prostate Imaging Reporting and Data System (PI-RADS) v2.1 protocol, all patients’ PI-RADS scores were derived from mpMRI images. The PI-RADS scores of PIN were obtained according to the location of PIN foci on the histopathological maps. The highest PI-RADS scores were recorded for BPH patients. The PV was calculated according to the following equation: PV = [maximum antero-posterior diameter (APD)] × [maximum longitudinal diameter (LD)] × [maximum transverse diameter (TD)] × 0.52. According to the PI-RADS v2.1 protocol, the maximum LD and maximum APD should be measured on the midsagittal T_2_WI, whereas the maximum TD should be measured on the transversal T_2_WI. An apparent diffusion coefficient (ADC) map of each patient was automatically generated from the DW images of all b values on the scanner. The DCE-MRI data were sent to a built-in workstation for postprocessing *via* the DCE-MRI software package. The DCE-MRI quantitative parameters [volume transfer constant (*K*^trans^), reflux rate constant (*k*_ep_), volume fraction of the extravascular extracellular space (*v*_e_), and initial area under the curve (iAUC)] were calculated by fitting the standard Tofts linear model to the concentration‒time curves. The regions of interest (ROIs) of the ADC, *K*^trans^, *k*_ep_, *v*_e,_ and iAUC were manually drawn according to the shape and size of the lesions on the T_2_WIs. The placement of ROIs and the evaluation of the mpMRI images were performed by an abdominal radiologist with 18 years of MRI experience. Special attention was paid to avoid necrosis, cystic areas, or vessels during the measurement process. The average value of three measurements of each parameter was used for the final result.

### 2.3 Statistical Analysis

All data analyses were carried out by using SPSS Statistics software for Windows (Version 25.0, SPSS, Inc., Chicago, IL) and MedCalc software (Version 12.3, MedCalc Software, Mariakerke, Belgium). Parameter values are presented herein as the means ± standard deviations for normally distributed variables and as medians with quartiles for skewed distributed variables. The data distribution was evaluated *via* the Shapiro‒Wilk test, normally distributed variables were compared *via* Student's *t* test, and the skewed distributed variables were compared *via* the nonparametric Mann‒Whitney U test. Receiver operating characteristic (ROC) curves were analysed to evaluate the diagnostic performance and find the optimal threshold value. Multivariate logistic regression analysis was conducted to identify the independent variables, and variables with a univariable value of *P*-value < 0.05 were incorporated into the multivariable model. Two-tailed *P* values < 0.05 were considered a statistically significant difference.

## 3 RESULTS

### 3.1 Comparison of mpMRI- and PSA-derived Parameters

A comparison of mpMRI- and PSA-derived parameters between BPHs and PINs is presented in Table **2**.

**Fig. (2):**
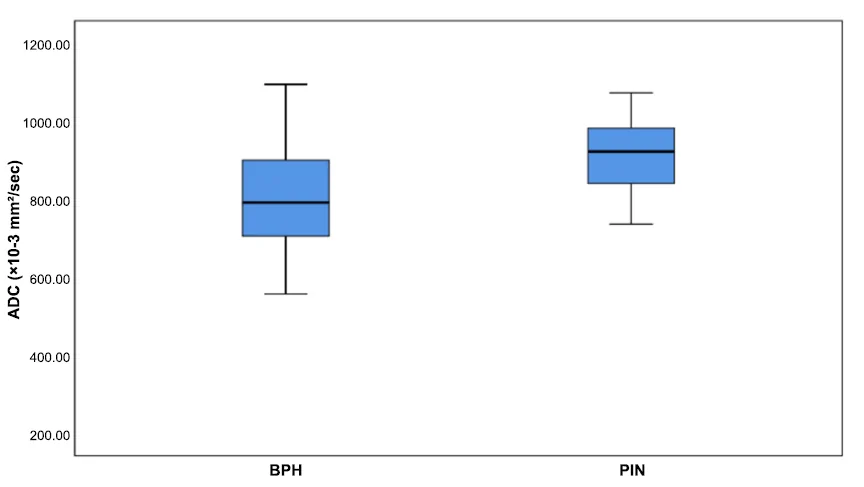
Box and whisker plots compared the ADC value between PIN and BPH. This line stands for the median. The central box stands for the measurements from the lower (the 25th percentile) to the upper (the 75th percentile) quartiles. Whiskers manifest the range from the minimal to the maximal parameter measurements. This plot revealed that the ADC of PIN was significantly greater than that of BPH. ADC, apparent diffusion coefficients; BPH, benign prostatic hyperplasia; PIN, prostatic intraepithelial neoplasia.

The ADC value of PIN was significantly greater than BPH (0.912 × 10^-3^ *vs.* 0.806 × 10^-3^ mm^2^/s, *P*=0.035) (Fig. **2**). There were no significant differences in terms of the *K*^trans^, *k*_ep_, *v*_e_, or iAUC values between patients with PIN and those with BPH (*P* > 0.05; for all comparisons).

**Fig. (3):**
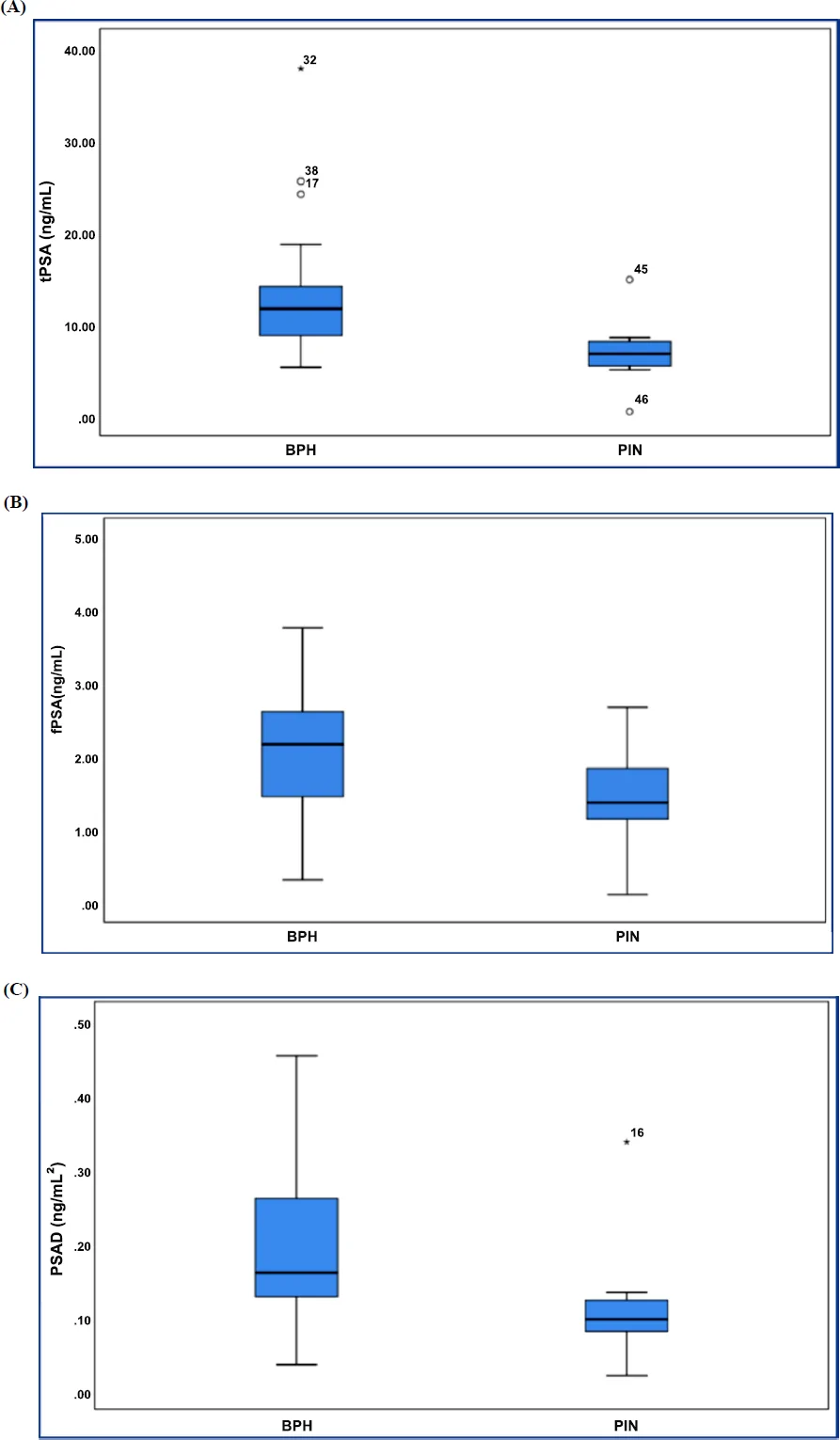
Box and whisker plots comparing the tPSA (**A**), fPSA (**B**), and PSAD (**C**) between PIN and BPH. This line stands for the median. The central box stands for the measurements from the lower (the 25th percentile) to the upper (the 75th percentile) quartiles Whiskers manifest the range from the minimal to the maximal parameter measurements. These plots revealed that the tPSA, fPSA, and PSAD of PIN was significantly lower than those of BPH. BPH, benign prostatic hyperplasia; fPSA, free prostate specific antigen; PIN, prostatic intraepithelial neoplasia; PSAD, prostate specific antigen density; tPSA, total prostate specific antigen.

The tPSA and fPSA of PIN were significantly less than BPH (6.715 *vs.* 11.640 ng/mL, *P*=0.008; 1.427 *vs.* 2.109 ng/mL, *P*=0.042, respectively) (Fig. **3A**, ****B****). The PSAD of PIN was significantly less than BPH (0.095 *vs.* 0.158 ng/mL^2^, *P*=0.010) (Fig. **3C**). There were no significant differences in terms of the f/tPSA or PV between BPHs and PINs (*P* > 0.05; for all comparisons).

### 3.2 ROC and Multivariate Analysis of Clinical Characteristics and mpMRI Parameters

The ROC analysis results of the clinical characteristics and mpMRI parameters for differentiating PIN from BPH are shown in Table **3**. The area under the curve (AUC) of tPSA was slightly greater than those of PSAD, ADC, and fPSA (0.801 >0.792 > 0.724 > 0.716) in differentiating PIN from BPH. When tPSA was ≤ 8.47 ng/mL, the highest sensitivity (87.5%) and negative predictive value (NPV, 96.8%) were observed for the differentiation of PIN from BPH; however, the specificity (76.9%) and positive predictive value (PPV, 43.8%) were relatively low. When the ADC and PSA-derived parameters were combined, the highest specificity (94.9%) and positive predictive value (PPV, 66.7%) were acquired in differentiating PIN from BPH. The above corresponding ROC curve map is shown in Fig. (**4A**-**E**).

Multivariate analysis revealed that ADC [odds ratio (OR)=0.120, 95% confidence interval (CI)=0.015 - 0.934; *P* = 0.043)] and PSAD [odds ratio (OR)=0.056, 95% confidence interval (CI)=0.007 - 0.427; *P* = 0.005)] were independent risk predictors for differentiating PIN from BPH. Multivariate analysis results are presented in Table **4**.

## 4 DISCUSSION

### 4.1 PSA-derived Parameters in Differentiating PIN from BPH

This study revealed significant statistical differences in tPSA, fPSA, and PSAD between patients with BPH and those with PIN. Serum PSA is the most commonly used bioindicator for detecting early-stage PCa. PIN is a transitional state between normal status and malignancy in prostate carcinogenesis [13], and it may precede clinically detected PCa by several decades [5, 6]. HGPIN may be an appropriate biomarker for latent PCa, and LGPIN may also be closely associated with PCa [4]. Timely detection of these precancerous processes can improve the early diagnosis and treatment of PCa. Radiation therapy and androgen deprivation therapy decrease the morbidity and degree of PIN and result in the regression of HGPIN [14, 15]. Therefore, early detection of PIN is clinically crucial for the prevention of PIN occurrence and malignancy. Unlike the findings from the previous study [16], this study disclosed that the tPSA and fPSA levels of PIN patients were markedly lower than those of BPH patients. This may be because the PV of BPH patients was greater than that of PIN patients in our study. The larger PV of BPH may account for the higher serum tPSA and fPSA levels of BPH than those of PIN [30]. Previous research [20] reported that fPSA was increased in BPH and decreased in PCa, as our research results exhibited that the fPSA of PIN was markedly lower than that of BPH. Research on fPSA might be very important, especially for distinguishing between BPH patients and PIN patients. PASD can decrease the impact of enlarged PVs [19]. The present study revealed that the PASD of PIN patients was markedly less than that of BPH patients. However, because the study cohorts of this research were small, these results need to be further confirmed in a larger patient population.

### 4.2 mpMRI Parameters in Differentiating PIN from BPH

The results of this study demonstrated that the average ADC value of PIN was notably greater than that of BPH. The texture of BPH is heterogeneous. The cytomorphological features of BPH include abundant fibrillar connective tissue containing many smooth muscle cells, fibroblasts, and myofibroblasts between the small glands [31]. The characteristic of abundant cells and fibres may cause the decreased ADC value of BPH. The structural features of PIN include the proliferation of secretory cells within preexisting ducts and acini, accompanied by cytological atypia [4]. The basal cell layer is intact in LGPIN or partly interrupted in HGPIN [7]. The excretions of proliferative secretory cells increase the water content within the ducts and acini, which may result in the increased diffusion of water molecules in PIN tissues compared with that in BPH tissues. The PIN patients tend to have higher ADC values. Because of partial-volume effects and ROI placement challenges in PIN, especially with small lesions, the results of this study may be inaccurate. This result needs to be further confirmed in studies with a larger patient population.

### 4.3 The Diagnostic Efficacy of mpMRI and PSA-derived Parameters in Differentiating PIN

This study revealed that tPSA had the highest diagnostic efficiency in discriminating PIN from BPH (AUC, 0.801), followed by PSAD, ADC, and fPSA (AUC, 0.792, 0.724, and 0.716, respectively). The highest sensitivity and NPV were achieved for tPSA levels of ≤ 8.47 ng/mL when differentiating PIN from BPH; however, the specificity and PPV were relatively low. When the ADC and PSA parameters (tPSA, fPSA, and PSAD) were combined, the highest specificity and PPV were obtained in the differentiation of PIN from BPH. The higher specificity for differentiating PIN from BPH could avoid an unnecessary prostate biopsy during the management of prostatic patients. Therefore, the ADC derived from mpMRI combined with PSA parameters may be a potential screening indicator for differentiating PIN from BPH. Multivariate logistic regression analysis revealed that ADC and PSAD could be used as the independent prognostic factors for differentiating PIN from BPH (OR=0.120, *P* = 0.043; OR=0.056, *P* = 0.005).

### 4.4 Study Limitations and Future Directions

This study had several limitations. First, the small number of subjects was a limitation of this study, especially regarding the limited number of PIN cases. Further studies with a larger patient population are needed to assess the mpMRI characteristics of PIN. Second, it was difficult to correlate the location of PIN in mpMRI images and histopathological maps very precisely, although standard 12-core TRUS-guided biopsies were performed. Third, our study subjects were not divided into HGPIN and LGPIN groups because of the small number of PIN cases. Research concerning LGPIN, HGPIN, and BPH will be assessed in further studies with a larger patient population.

## CONCLUSION

The ADC value and PSA-derived parameters (tPSA, fPSA, and PSAD) can distinguish PIN from BPH. The ADC value combined with the tPSA level can be used to determine the highest diagnostic efficiency in detecting PIN. The ADC derived from mpMRI combined with serum PSA-derived parameters may function as a potentially valuable biomarker to differentiate precancerous lesions of the prostate at earlier stages.

## Figures and Tables

**Fig. (1):**
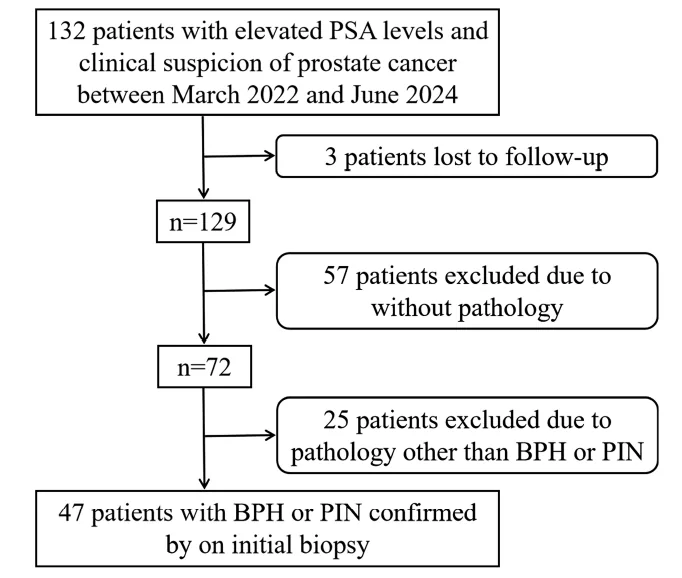
Diagram of study population. The flowchart shows the patient's selection process. Forty seven of all the 132 patients were included in present study. BPH, benign prostatic hyperplasia; PIN, prostatic intraepithelial neoplasia; PSA, prostate specific antigen.

**Fig. (4A-E):**
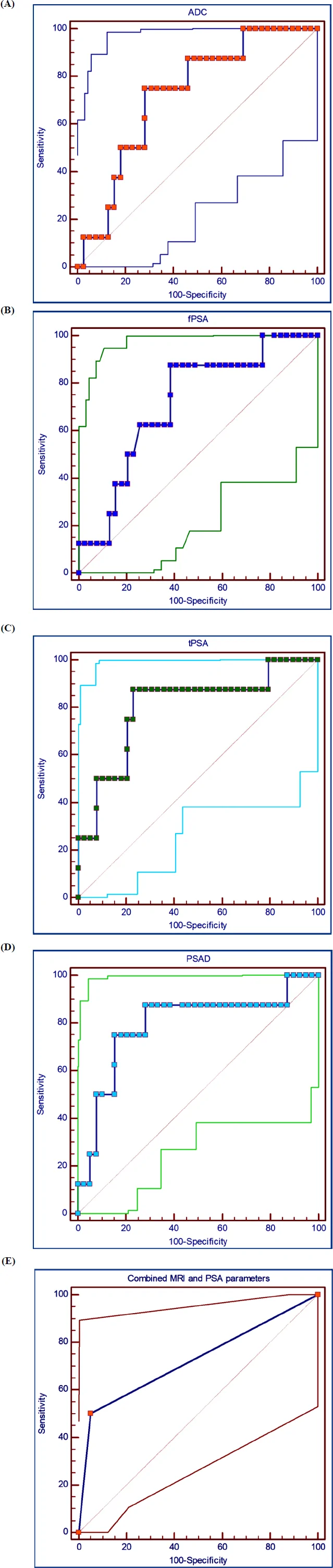
ROC curves of mpMRI and PSA-derived parameters for distinguishing between PIN and BPH. The diagonal line stands for the reference line which manifests the results for a test with 50% specificity and 50% sensitivity. This figure showed that the greatest AUC can be discovered for tPSA, and followed by PSAD, ADC and fPSA (0.801 > 0.792 > 0.724 > 0.716) in differentiating PIN from BPH. AUC, area under curve; BPH, benign prostatic hyperplasia; PIN, prostatic intraepithelial neoplasia; ROC, receiver operating characteristic; tPSA, total prostate specific antigen.

**Table 1:** Acquisition parameters of the mpMRI protocol.

-	TR (ms)	TE (ms)	FOV (cm)	Slice Thickness (mm)	Slice Gap (mm)	Matrix	Number of Excitation	Bandwidth (Hz/pixel)	Acquisition Time (sec)
Sagittal T_2_WI	8960	122	20×20	3.0	0.3	320×256	2	504	134
Coronal T_2_WI	5000	106	20×20	3.0	0.3	320×256	2	391	125
Axial T_2_WI	4870	87	20×20	3.0	0.3	320×256	2	313	131
Axial T_1_WI	500	9.4	34×34	5.0	1.5	448×358	1	302	125
DWI	5600	73	30×30	3.0	0.3	114×114	1~6	2088	201
Axial T_1_WI (VIBE)	5.08	1.77	26×26	3.5	0.7	192×154	1	260	9.5
Axial DCE-MRI (VIBE)	5.08	1.77	26×26	3.5	0.7	192×154	1	260	332

**Abbreviations:** DCE-MRI, dynamic contrast enhanced magnetic resonance imaging; DWI, diffusion weighted imaging; FOV, field of view; mpMRI, multiparametric magnetic resonance imaging; TE, Echo time; TR, Repetition time; TSE, turbo spin echo; T_1_WI, T1 weighted imaging; T_2_WI, T2 weighted imaging; VIBE, volume interpolated body examination.

**Notes:** Specific number of excitation for each b-value is listed as follows: 50(1), 500(1), 1000(4), 1500(6) sec/mm^2^. The numbers in brackets represent number of excitation.

**Table 2:** Comparison of mpMRI and PSA parameters between BPH and PIN.

Parameter	BPH (39)	PIN (8)	t/Z*-*Value	*p*-value
ADC (×10^-3^ mm^2^/s)	0.806 ± 0.157	0.912 ± 0.107	2.348	0.035
*K*^trans^ (min^-1^)	0.183 (0.090-0.344)	0.101 (0.075-0.539)	0.451	0.652
*k*_ep_ (min^-1^)	0.472 (0.343-0.891)	0.401(0.299-1.490)	0.367	0.713
*v*_e_	0.297 (0.245-0.514)	0.266 (0.224-0.431)	0.835	0.404
iAUC	13.205 (8.383-25.052)	11.334 (9.704-11.361)	0.033	0.973
tPSA (ng/mL)	11.640 (8.640-14.130)	6.715 (5.175-8.275)	2.661	0.008
fPSA (ng/mL)	2.109 ± 0.923	1.427 ± 0.739	2.270	0.042
f/tPSA (%)	18.308 ± 7.168	21.500 ± 3.295	1.952	0.063
PSAD (ng/mL^2^)	0.158 (0.124-0.278)	0.095 (0.075-0.126)	2.576	0.010
PV (cm^3^)	74.20 (51.200-95.330)	66.565 (30.648-91.560)	0.651	0.515

**Abbreviations:** ADC, apparent diffusion coefficients; BPH, benign prostatic hyperplasia; fPSA, free prostate specific antigen; f/tPSA, free/total prostate specific antigen; iAUC, initial area under curve; *k*_ep_, reflux rate constant; *K*^trans^, volume transfer constant; mpMRI, multiparametric magnetic resonance imaging; PIN, prostatic intraepithelial neoplasia; PSAD, prostate specific antigen density; PV, prostate volume; tPSA, total prostate specific antigen; *v*_e_, volume fraction of the extravascular extracellular space.

**Table 3:** Results of the ROC analyses.

-	**AUC (95% CI)**	**Cut-off Value**	**Sensitivity (%)**	**Specificity (%)**	**PPV (%)**	**NPV (%)**	***p*-value**
mpMRI parameter	-	-	-	-	-	-	-
ADC	0.724 (0.575-0.845)	> 0.875 ×10^-3^ mm^2^/s	75.0	71.8	35.3	93.3	0.013
PSA parameters	-	-	-	-	-	-	-
tPSA	0.801 (0.659-0.903)	≤ 8.47 ng/mL	87.5	76.9	43.8	96.8	0.002
fPSA	0.716 (0.566-0.838)	≤ 1.88 ng/mL	87.5	61.5	31.8	96.0	0.024
PSAD	0.792 (0.648-0.896)	≤ 0.11 ng/mL^2^	75.0	84.6	50.0	94.3	0.006
Combined ADC and PSA parameters	0.724 (0.575-0.845)	-	50.0	94.9	66.7	90.2	0.020

**Abbreviations:** ADC, apparent diffusion coefficients; AUC, area under curve; fPSA, free prostate specific antigen; mpMRI, multiparametric magnetic resonance imaging; NPV, negative predictive value; PPV, positive predictive value; PSA, prostate specific antigen; PSAD, prostate specific antigen density; ROC, receiver operating characteristic; tPSA, total prostate-specific antigen.

**Table 4:** Multivariate logistic regression analysis for detecting PIN.

**Parameter**	**OR**	***p*-Value**	**95% CI**
ADC (×10^-3^ mm^2^/s)	0.120	0.043	0.015 - 0.934
PSAD (ng/mL/cm^3^)	0.056	0.005	0.007 - 0.427

**Abbreviations:** ADC, apparent diffusion coefficients; CI, confidence interval; PIN, prostatic intraepithelial neoplasia; PSAD, prostate specific antigen density; OR, odds ratio.

## Data Availability

All datasets presented in this study are included in the article. Further inquiries can be directed to the corresponding authors [X.L] and [W.M].

## LIST OF ABBREVIATIONS

ADC: Apparent Diffusion Coefficients
APD: Antero-Posterior Diameter
AUC: Area Under Curve
BPH: Benign Prostatic Hyperplasia
CI: Confidence Interval
DCE: Dynamic Contrast Enhanced
DRE: Digital Rectal Examination
DWI: Diffusion-Weighted Imaging
fPSA: Free PSA
f/tPSA: Free/Total Prostate Specific Antigen
HGPIN: High Grade Prostatic Intraepithelial Neoplasia
iAUC: Initial Area Under Curve
k_ep_: Reflux Rate Constant
K^trans^: Volume Transfer Constant
LD: Longitudinal Diameter
LGPIN: Low Grade PIN
MRSI: Magnetic Resonance Spectroscopic Imaging
mpMRI: Multiparametric Magnetic Resonance Imaging
NPV: Negative Predictive Value
OR: Odds Ratio
PCa: Prostate Cancer
PIN: Prostatic Intraepithelial Neoplasia
PI-RADS: Prostate Imaging Reporting And Data System
PPV: Positive Predictive Value
PSA: Prostate Specific Antigen
PSAD: PSA Density
PV: Prostate Volume
ROC: Receiver Operating Characteristics
ROIs: Regions Of Interest
TD: Transverse Diameter
tPSA: Total PSA
TRUS: Transrectal Ultrasound
T_1_WI: T1 Weighted Imaging
T_2_WI: T2 Weighted Imaging
V_e_: Volume Fraction Of The Extravascular Extracellular Space
3D-VIBE: 3D Volume Interpolated Body Examination

## STANDARDS OF REPORTING

STROBE guidelines were followed.
